# Homebirth and homecare during COVID-19

**DOI:** 10.18332/ejm/120972

**Published:** 2020-04-29

**Authors:** Revekka Ziogou, Katerina Zografou

**Affiliations:** 1 National Public Health Organization-PHILOS, Refugee Center, Alexandria, Greece; 2 Department of High-Risk Pregnancies and Postpartum Ward, General University Hospital of Patras, Patra, Greece

**Keywords:** homebirth, telecare, midwife, perinatal care, COVID-19, virtual visit

**Dear Editor,**

In the middle of the COVID-19 pandemic, even though a disease should be the number one reason to be admitted to a hospital, it turns out to be childbirth, in which midwives play an important role^1,2^. Within the COVID-19 pandemic, a pregnant woman who had planned to give birth at the hospital may have second thoughts and may get stressed about giving birth in such a hospital environment. Prenatal anxiety and depression are associated with preterm births, low birthweight babies, and small head circumference^3^.

Due to COVID-19, perinatal care has to be tailored to provide women with the best and most safe care. Midwives should offer alternatives to sustain and protect maternal and neonatal health. First, telecare is an excellent option during the perinatal period, which results in a reduction of face-to-face visits^4^ and minimizes the cost of care^5^. Second, blood pressure, weight and fetal heart rates can be remotely monitored^5,6^. Information and education can also be provided through telecare. Pregnant women are familiar with the use of smartphones and technology, and they have a positive attitude towards remote home-monitoring^7^.

A planned homebirth with a registered midwife could be an excellent and safe alternative for low-risk pregnant women. It is very important for both pregnant woman and midwife to have a birth plan, instead of a last-minute decision, as the transfer to the nearest hospital has to be prearranged, in the rare case that something goes wrong. Fewer interventions, including episiotomy, epidural anaesthesia or delivery with a vacuum take place during a homebirth compared to planned hospital births^8^. The comfort of home, the right to choose and the continuity of care are some of the main reasons why mothers choose to give birth at home. Moreover, the benefit from their partner’s presence and support are also very important^9^. During the postpartum period, a midwife may perform virtual or in-person home visits to check on mothers’ and infants’ health. Furthermore, home birth may cost less than a hospital birth^10^. Hospital and pharmaceutical expenses both for mother and infant are less when a woman delivers at home with a midwife.

Humanity has suffered many pandemics throughout its history, and this will probably not change in the future. As the Word Health Organization has declared 2020 the year of midwives, it is an excellent opportunity to establish a midwifery-led model of maternity care, which will minimize the cost and provide all women with high-quality perinatal care.
